# Residual Spinal Cord Compression Following Hemilaminectomy and Mini-Hemilaminectomy in Dogs: A Prospective Randomized Study

**DOI:** 10.3389/fvets.2017.00042

**Published:** 2017-03-23

**Authors:** Gustaf Svensson, Ulrika S. H. Simonsson, Fredrik Danielsson, Tobias Schwarz

**Affiliations:** ^1^Bla Stjarnans Djursjukhus, Gothenburg, Sweden; ^2^Department of Pharmaceutical Biosciences, Uppsala University, Uppsala, Sweden; ^3^Vetaid, Helsingborg, Sweden; ^4^Royal (Dick) School of Veterinary Studies, The University of Edinburgh, Roslin, Midlothian, UK

**Keywords:** hemilaminectomy, mini-hemilaminectomy, computed tomography, spinal cord compression, dogs

## Abstract

The aim of this study was to compare the reduction of spinal cord compression after surgical treatment of dogs with acute thoracolumbar intervertebral disc (IVD) extrusion achieved using hemilaminectomy versus mini-hemilaminectomy techniques. This was a prospective randomized study with client-owned dogs presented with acute IVD extrusion that were allocated to surgical treatment using hemilaminectomy (*n* = 15) or mini-hemilaminectomy (*n* = 15) techniques. Plain and intravenous-contrast computed tomography was performed pre- and postoperatively. The preoperative minimal cross-sectional dimension of the spinal cord (MDSC_pre_) and the postoperative minimal cross-sectional dimension of the spinal cord (MDSC_post_) were measured at the level of greatest compression. The minimal diameter of the uncompressed spinal cord was measured in a similar way both pre- (MDUSC_pre_) and postoperatively (MDUSC_post_). Dogs in the mini-hemilaminectomy group had significantly greater reduction of compression (RC) (*p* < 0.01) after surgery compared to dogs in the hemilaminectomy group. The mean RC in the hemilaminectomy group was 34.6% and in the mini-hemilaminectomy group 62.6%. Our results showed a significantly greater reduction of spinal cord compression for mini-hemilaminectomy compared to hemilaminectomy. Additionally, mini-hemilaminectomy could be a preferred method due to its minimal invasiveness and easier access to lateral fenestration.

## Introduction

Intervertebral disc (IVD) extrusion is a common disorder in dogs with a reported overall prevalence of 2–3.5% and a higher prevalence in chondrodystrophic dogs (1–3). Surgical decompression is a well-accepted treatment for dogs with neurological deficits secondary to IVD extrusion (4–6). Several different surgical methods have been described for the treatment of IVD extrusion and among them are hemilaminectomy and mini-hemilaminectomy (4–6). Mini-hemilaminectomy was first described by Jeffery and differs mainly from hemilaminectomy in two ways (7). First in the mini-hemilaminectomy procedure, the facet joint is not removed as it is using the hemilaminectomy technique. Second, lateral approach to the spine can be adopted when performing a mini-hemilaminectomy, which is less traumatic for the surrounding soft tissue structures (4). Also, fenestration through a lateral approach has been reported to lead to less residual nucleus material in the disc compared to a dorsal and dorsolateral approach (8). Other advantages with less invasive procedures such as the mini-hemilaminectomy are decreased vertebral instability and a faster recovery (7, 9, 10). The increased instability to the spinal segment after a hemilaminectomy could enhance adjacent segment IVD degeneration and thus increase the risk of adjacent IVDs herniating. Although this has been demonstrated *in vitro*, clinical effects of instability have not been shown (10, 11).

Surgical decompression with removal of all herniated disc material is the goal of the surgical treatment. In work by Roach et al., where hemilaminectomy was followed by a computed tomography (CT) scan, all patients had residual disc material after surgery. Return of hind limb function occurred in all cases despite the presence of residual disc material (12).

The purpose of this study was to compare the reduction of spinal cord compression achieved by surgical treatment of IVD extrusion between hemilaminectomy and mini-hemilaminectomy, evaluated through pre- and postoperative CT scans.

## Materials and Methods

### Animals

Client-owned dogs admitted to Blue Star Animal Hospital from January 2011 to December 2012, with acute thoracolumbar IVD extrusion with neurological grade 3 and 4 as classified by Toombs and Waters, were included in this study (13). Breed, sex, age, and weight were recorded preoperatively.

Exclusion criteria were IVD protrusion, history of back pain or neurological deficits exceeding 3 weeks, myelogram or myelogram-CT, and serum creatinine above normal values. Dogs were randomized, by sealed envelopes, into 2 groups with 15 animals in each group. Group one (Group H) had hemilaminectomy and fenestration of at least the affected IVD performed, and group two (Group M) had mini-hemilaminectomy and fenestration of at least the affected IVD performed. This research was approved by the Swedish Regional Ethical Review Board in Gothenburg. All animals in the study were clinical cases where clients volunteered their dog for the study and provided written consent.

### Computed Tomography

All dogs had pre- and post-intravenous (IV) contrast CT studies of diagnostic quality performed both pre- and postoperatively. The postoperative CT was covering the area of spine decompressive surgery and at least two vertebral body lengths on cranially and caudally. All preoperative CTs were covering a larger area than the postoperative CTs. Dogs were anesthetized and positioned in dorsal recumbency. Anesthesia was induced intravenously with acepromazine (0.02 mg/kg, Plegicil^®^, Pharmaxim, Helsingborg, Sweden), methadone (0.04 mg/kg, Metadon Recip^®^, Recip, Solna, Sweden), and propofol (4 mg/kg, Propovet^®^, Orion Pharma, Sollentuna, Sweden) and maintained on sevoflurane (Sevoran^®^, AbbVie, Solna, Sweden) and a continuous rate infusion of morphine (0.25 mg/kg/h, Morfin Meda^®^, Meda, Solna, Sweden), ketamine (0.6 mg/kg/h, Ketaminol^®^, Intervet, Sollentuna, Sweden), and lidocaine (3 mg/kg/h, Xylocain^®^, AstraZeneca, Sodertalje, Sweden). CT scanning was performed with a 16-slice helical CT (Philips Brilliance^®^, Philips Healthcare, Stockholm, Sweden). Constant CT parameters included 120 kV tube voltage, adaptive tube current 200–300 mA, tube rotation time 1 s, helical acquisition mode, bone and soft tissue kernel image reconstructions, 1-mm slice thickness, 0.5-mm image reconstruction interval, and a collimator pitch of 0.817. IV iodine contrast medium (Omnipaque^®^, GE Healthcare, Stockholm, Sweden) was administered *via* power injector at a dose of 700 mg/kg. Post contrast scanning commenced within 1 min post start injection. Between the day of surgery and 7 days after, another CT scan was performed to assess the degree of remaining spinal cord compression. The CT scan was performed in the same way as preoperatively. Dogs that did not have their second CT direct after surgery were sedated with dexmedetomidine (0.01 mg/kg, Dexdomitor^®^, Orion Pharma, Stockholm, Sweden) and butorphanol (0.3 mg/kg, Dolorex vet^®^, Intervet, Sollentuna, Sweden).

The vertebral canal was aligned properly with the *Z*-axis and *X*–*Y* axes of the CT scanner, so that the original transverse CT images represented the transverse plane of the vertebral canal at the laminectomy surgery site.

All measurements were generated from the original transversely oriented CT images on a dedicated DICOM work station (Osirix^®^ 3.9.2. 64 bit, Pixmeo, Bernex, Switzerland) using a calibrated monitor (Apple^®^ 30 inch Cinemax display, Apple Inc., Cupertino, CA, USA). The image evaluation was performed by a single boarded veterinary radiologist (Tobias Schwarz). First the laminectomy site, side, and extension were recorded based on multiplanar reconstruction images of bone image reconstruction kernel postoperative images. Pre- and postoperative post contrast CT series were displayed side by side with a standardized window setting (window level 70 HU, window width 170 HU).

Slight windowing adjustments for optimal lesion visualization were permitted, but both series were always displayed with the same setting. The image location in relation to the vertebral anatomy of both series was manually synchronized using bony landmarks. The linear measurement tool was used for distance measurements.

**Figure 1:**
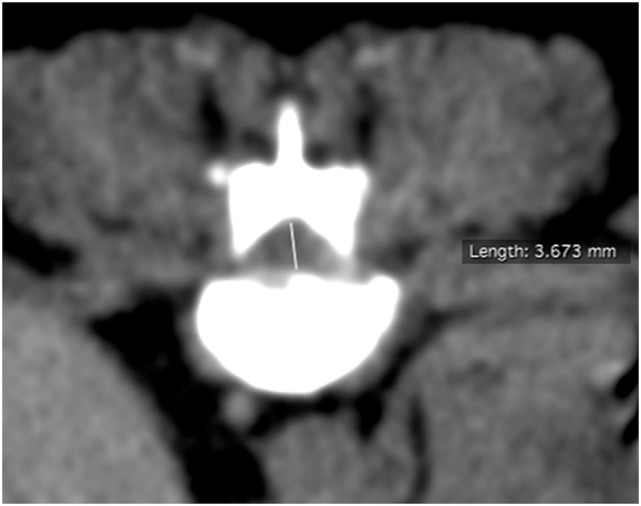
**Preoperative transverse computed tomography image at site of maximal compression with measurement of minimal cross-sectional dimension**.

**Figure 2:**
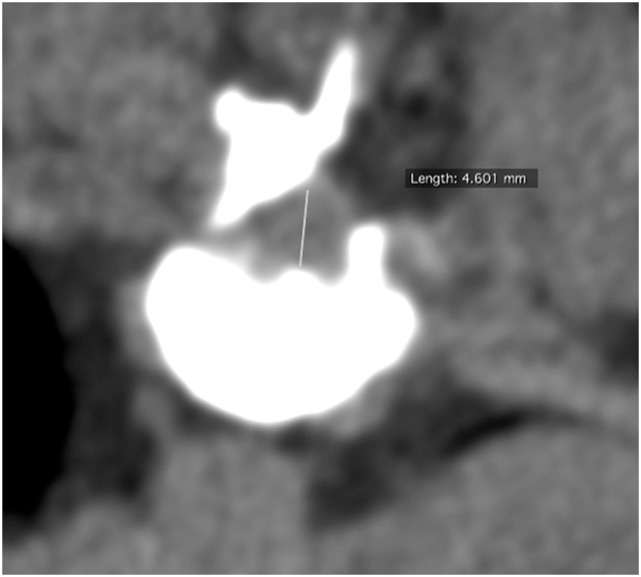
**Postoperative (hemilaminectomy) transverse computed tomography image at site of maximal compression with measurement of minimal cross-sectional dimension**.

The preoperative minimal cross-sectional dimension of the spinal cord (MDSC_pre_) at level of greatest compression within the range of the laminectomy site was measured as well as the postoperative minimal cross-sectional dimension of the spinal cord (MDSC_post_) at level of greatest compression (see Figures 1 and 2). The cross-sectional measurement had to cross the isocenter of the spinal cord and could not be tangential. The exact slice location (Z-axis location) at which the measurement was taken was recorded for both series.

### Surgical Techniques

All surgeries were performed by a board certified surgeon (Fredrik Danielsson) or last year resident (Gustaf Svensson), both experienced neurosurgeons. Dogs randomized into Group H had hemilaminectomy through a dorsolateral approach performed as previously described (5, 6, 14). Dogs randomized into Group M had mini-hemilaminectomy performed as described in the original paper by Jeffery with a lateral approach (7, 14). Location and length of the laminectomy was recorded. The length of the laminectomy was based on CT evaluation and/or until normal epidural fat was visible in the laminectomy. All dogs had the affected disc fenestrated, spinal canal checked after fenestration for disc material, and a fat graft placed over the laminectomy. According to the surgeon, all visible disc material was removed in all cases.

### Statistical Analysis

Reduction of compression (RC) in each animal between pre- and postoperation was defined as:

$$\text{RC}=\frac{100\left({\text{MDSC}}_{\text{post}}-{\text{MDSC}}_{\text{pre}}\right)}{{\text{MDSC}}_{\text{pre}}}.$$

The geometric mean and 95% confidence interval (CI; obtained from ln-transformed data) of MDSC_pre_, MDSC_post_, and RC were calculated for each group. The difference in compression between pre- and postoperation (MDSC_pre_ versus MDSC_post_) was evaluated in each study group using a paired *t*-test. In addition, the difference in RC between the two surgery methods was evaluated using an independent *t*-test. *T*-tests were done using ln-transformed data.

## Results

### Animals

The 30 dogs included the following breeds; Dachshund (*n* = 12), mixed breed (*n* = 7), Cavalier King Charles Spaniel (*n* = 1), French Bulldog (*n* = 1), Hamilton Hound (*n* = 1), Danish Swedish Farm dog (*n* = 1), Jack Russell Terrier (*n* = 1), Cocker Spaniel (*n* = 1), Drever (*n* = 1), Japanese Chin (*n* = 1), Border Terrier (*n* = 1), Pekingese (*n* = 1), and Shih Tzu (*n* = 1). Distribution with respect to number of chondrodystrophic dogs, sex, age, and weight are shown in Table 1. The mean number of days to postop CT was 3.3 (0–7) and 3.0 (0–6) in the hemilaminectomy group and in the mini-hemilaminectomy group, respectively. Five of the dogs in Group H and six in Group M had their postoperative CT immediately after surgery.

**Table 1:** **Descriptive statistics of demographics in hemilaminectomy group (Group H) and mini-hemilaminectomy group (Group M)**.

	Chondrodystrophic	Males	Females	Age (years)	Weight (kg)
Total	73.3%	20	10		
Mean				7.4	10.8
Median				8.0	9.6
Range				2.0–11.0	3.8–29.0
Group H	66.6%	10	5		
Mean				7.7	11.8
Median				8.0	9.0
Range				4.0–11.0	6.2–29.0
Group M	80.0%	10	5		
Mean				7.1	9.8
Median				8.0	9.6
Range				2.0–11.0	3.8–18.0

### CT Findings

**Figure 3:**
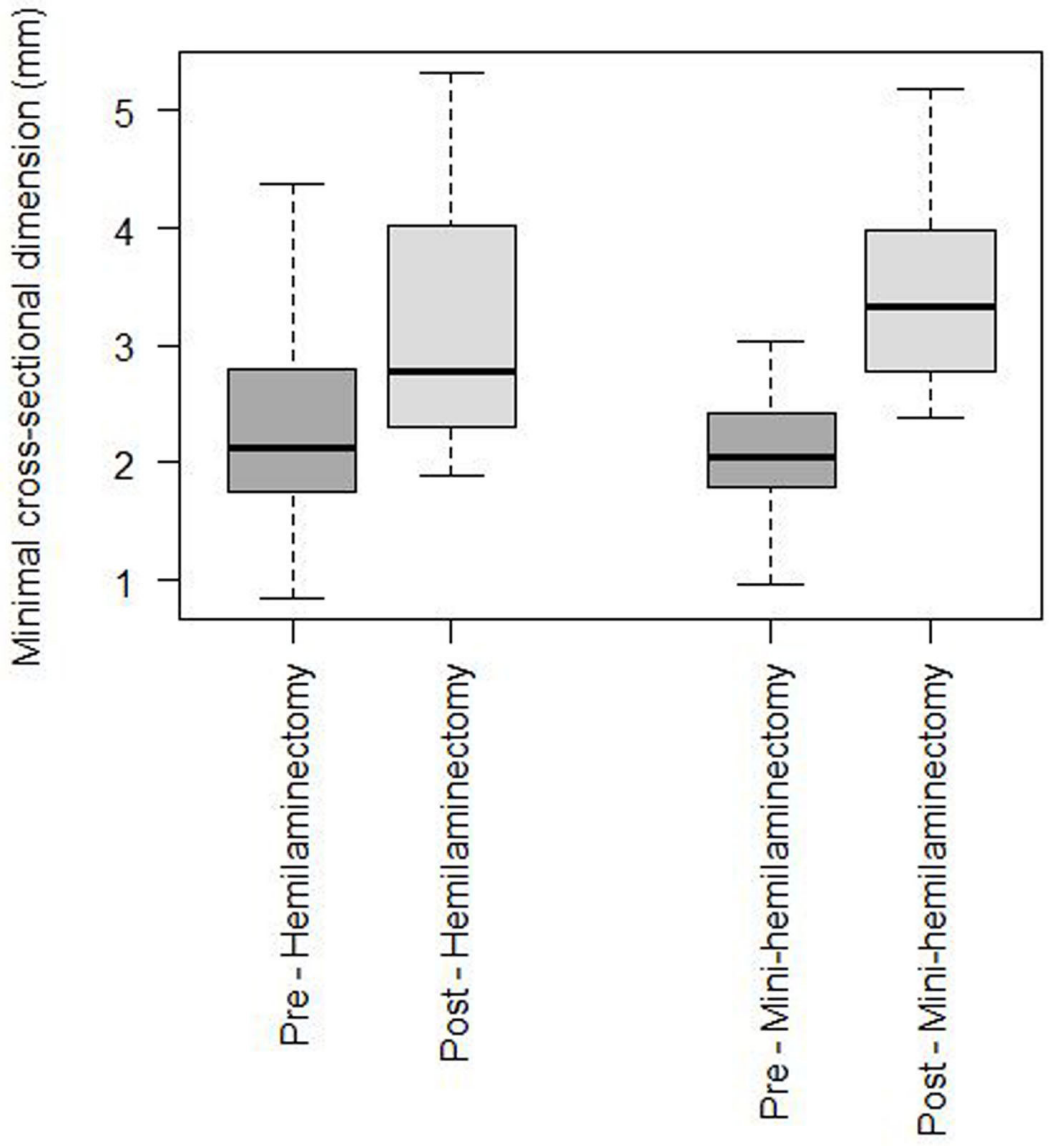
**Box and whisker plot of observed pre- and postoperative data for the hemilaminectomy group and mini-hemilaminectomy group**.

Surgery locations varied from T10 to L6, and the length of laminectomies varied from one to three levels. Mean length in Group H was 1.5 levels and in Group M 1.6 levels. The observed MDSC_pre_ and MDSC_post_ in the two treatment groups are shown in Figure 3. There was no significant difference in the compression preoperatively between the two groups. Descriptive statistics of MDSC_pre_ and MDSC_post_ in the hemilaminectomy group (Group H) and the mini-hemilaminectomy group (Group M) are shown in Table 2. The geometric mean MDSC_pre_ of the spinal cord in Group H was 2.18 mm. The geometric mean MDSC_post_ in Group H was 3.03 mm. There was a statistical significant difference between pre- and postoperative cross-section dimension of the spinal cord in Group H (*p* < 0.001). The geometric mean MDSC_pre_ and MDSC_post_ was 1.99 and 3.31 mm in Group M, respectively (*p* < 0.001).

**Table 2:** **Descriptive statistics of preoperative minimal cross-sectional dimension of the spinal cord (MDSC_pre_), postoperative minimal cross-sectional dimension of the spinal cord (MDSC_post_), and reduction of compression (RC)^a^ in dogs randomized to hemilaminectomy (Group H) and mini-hemilaminectomy (Group M)**.

Study group		MDSC_pre_ (mm)	MDSC_post_ (mm)	RC (%)^a,^*
Group H***	Geometric mean	2.18	3.03	27.2
	95% confidence interval (CI)^b^	0.94–3.42	1.84–4.23	−349
Group M***	Geometric mean	1.99	3.31	60.4
	95% CI^b^	0.82–3.15	2.18–4.44	−271

^a^$\text{RC}=\frac{100\left({\text{MDSC}}_{\text{post}}-{\text{MDSC}}_{\text{pre}}\right)}{{\text{MDSC}}_{\text{pre}}}.$

*^b^95% CI derived from ln-transformed data*.

*p < 0.05 between groups H and M.

***p < 0.001 between MDSC_pre_ and MDSC_post_.

Mean RC in group H was 27% and in Group M it was 60%. RC after mini-hemilaminectomy (Group M) was statistical significant greater (*p* < 0.05) compared to after hemilaminectomy (Group H).

## Discussion

To the author’s knowledge, this is the first time the residual spinal cord compression after hemilaminectomy and mini-hemilaminectomy has been compared in dogs. In our limited number of cases, we observed a significantly greater reduction of spinal cord compression after mini-hemilaminectomy compared to after hemilaminectomy. There was no significant difference in the compression preoperatively between the two groups. The mean preoperatively compression (MDSC_pre_) was 2.2 and 2.0 mm in dogs randomized to hemilaminectomy and mini-hemilaminectomy. Both surgical methods significantly reduced the compression postoperatively. However, greater RC after mini-hemilaminectomy (*p* < 0.05) was a surprising finding given that one would expect that a larger laminectomy defect would lead to at least equal amount of decompression, if not better, as compared to the mini-hemilaminectomy. The reason for the superior decompression achieved using the mini-hemilaminectomy could be due to improved access to the ventral aspect of the spinal canal and thereby more successful removal of extruded disc material.

Computed tomography with and without iodine IV contrast medium application was used to measure maximal compression of the spinal cord pre- and postoperatively with hemilaminectomy and mini-hemilaminectomy. CT has been proven in several studies to be a sensitive diagnostic tool for diagnosing canine IVD extrusion, both with and without IV contrast medium application (15–18). The use of myelogram-CT would probably have increased our sensitivity for detecting spinal cord compression, but it was difficult to justify a second myelogram after surgery considering the need for anesthesia instead of sedation and the risks involved with performing a myelogram. In a previous study where myelogram-CT showed the highest sensitivity, compared to IV contrast and plain CT, for detecting spinal cord compression a single-slice CT unit was used (15). In our study, a 16-slice CT unit was used, which probably increased our sensitivity. Dogs that needed myelogram-CT for an accurate diagnosis were excluded from the study since we did not perform myelogram-CT postoperatively. If myelogram-CT was needed for accurate diagnosis, plain and IV contrast images were not suitable for measuring spinal cord compression in this study. In this study single point measurement was used for evaluation of spinal cord compression as previously described (19, 20). We believe that it is still unproven if maximal compression, area of maximal compression, or volume compression is most important in the disease and therefore chose maximal compression since it is less prone to measurement errors. Single point measurements are, however, influenced by the difference in spinal cord and vertebral canal dimensions at different locations (21). The point of maximal compression can differ between pre- and postoperative CT examinations, and the same amount of disc material could at different sites result in different compression. Although this might be a confounder, we believe this potential difference is the same for both surgical techniques.

Mini-hemilaminectomy has been described using both a lateral and a dorsal approach to the vertebrae lamina (5, 7). We used a lateral approach for two reasons. First, we believe it is easier to visualize and evacuate disc material ventral to the spinal cord with minimal manipulation. Second, it made fenestration through a lateral approach easier. Fenestration reduces the recurrence of IVD extrusion after decompressive surgery (22, 23). A lateral approach to the IVD lead in a cadaveric study to less residual nucleus material in the disc compared to a dorsal and dorsolateral approach (8). This approach makes it also easier to reach the contralateral side of the disc where most of the remaining nucleus is located as fenestration is mostly performed on the same side as the extrusion. Furthermore, the trauma to surrounding structures is reduced with a lateral approach since less muscle tissue is disrupted and the facet joint is left in place (8).

The importance of remaining compression postoperative is unclear. Remaining disc material did not appear to affect the clinical outcome in the study by Roach et al. where all dogs were ambulatory at long-term follow-up (12). However, in another study with 19 dogs with follow-up by magnetic resonance imaging (MRI) postoperatively, only 1 dog had remaining disc material postoperatively. MRI done at 6 weeks postoperatively in the same study showed that 10 dogs had recurrence of extrusion and 3 of those dogs had clinical signs of disease (23). In this study, all cases but one had still compression left on the spinal cord after surgery, although since compression was measured instead of remaining disc material postoperatively, compression postoperatively could come from either disc material, hemorrhage, or fat graft.

Limitations of this study are that the design did not allow blinding of the reviewer toward the fact that there was a laminectomy performed. More importantly, since the measurement had to be performed at the exact same vertebral location, it was not possible to anonymize and randomize the CT studies. However, the measurement rules were set very strict to minimize any subjective influence. For instance, size measurements of anatomic structures in CT are greatly influenced by the point spread function, which is dependent on the window settings (24). This was standardized in our study to eliminate point spread function as a source of measurement error. Repeated measurements could have been helpful for intra- and inter-observer variations. However, this was beyond the scope of this study. Quantification of canine spinal cord compression has been performed in several studies using different methodologies, often with conflicting results (20, 25–27). Another limitation is the fact that due to practical reasons all dogs did not have their postoperative CT done at the same time; however, the mean number of days to postoperative CT was similar between Group H (3.3 days) and Group M (3.0 days).

In conclusion, our results in this limited number of cases indicate that mini-hemilaminectomy results in a significantly larger reduction of spinal cord compression compared to after hemilaminectomy. If these results are confirmed, mini-hemilaminectomy could be a preferred method over hemilaminectomy in dogs with IVD extrusion due to its lesser invasiveness, lateral fenestration, and potentially greater spinal decompression.

## Author Contributions

GS wrote the majority of the manuscript, TS wrote parts all radiology parts, and all authors took part in editing the manuscript. GS, FD, and TS designed the study. GS and FD performed the surgeries, TS performed all CT measurements, and US analyzed all data and performed the statistical analysis.

## Conflict of Interest Statement

The research was conducted in the absence of any commercial or financial relationships that could be construed as a potential conflict of interest.
